# Supplementary material for: The interactome of intact mitochondria by cross-linking mass spectrometry provides evidence for coexisting respiratory supercomplexes
Source: Mol Cell Proteomics. 2017 Dec 8;17(2):216–32. doi: 10.1074/mcp.RA117.000470 (PMC5795388; doi:10.1074/mcp.RA117.000470)
Supplement: Supplemental Data [file supp_RA117.000470_133922_0_supp_23978_fzffwf.zip › spectra_annotation/mito_DR_spectra_annotation/199-1-15-1-5-1.pdf]

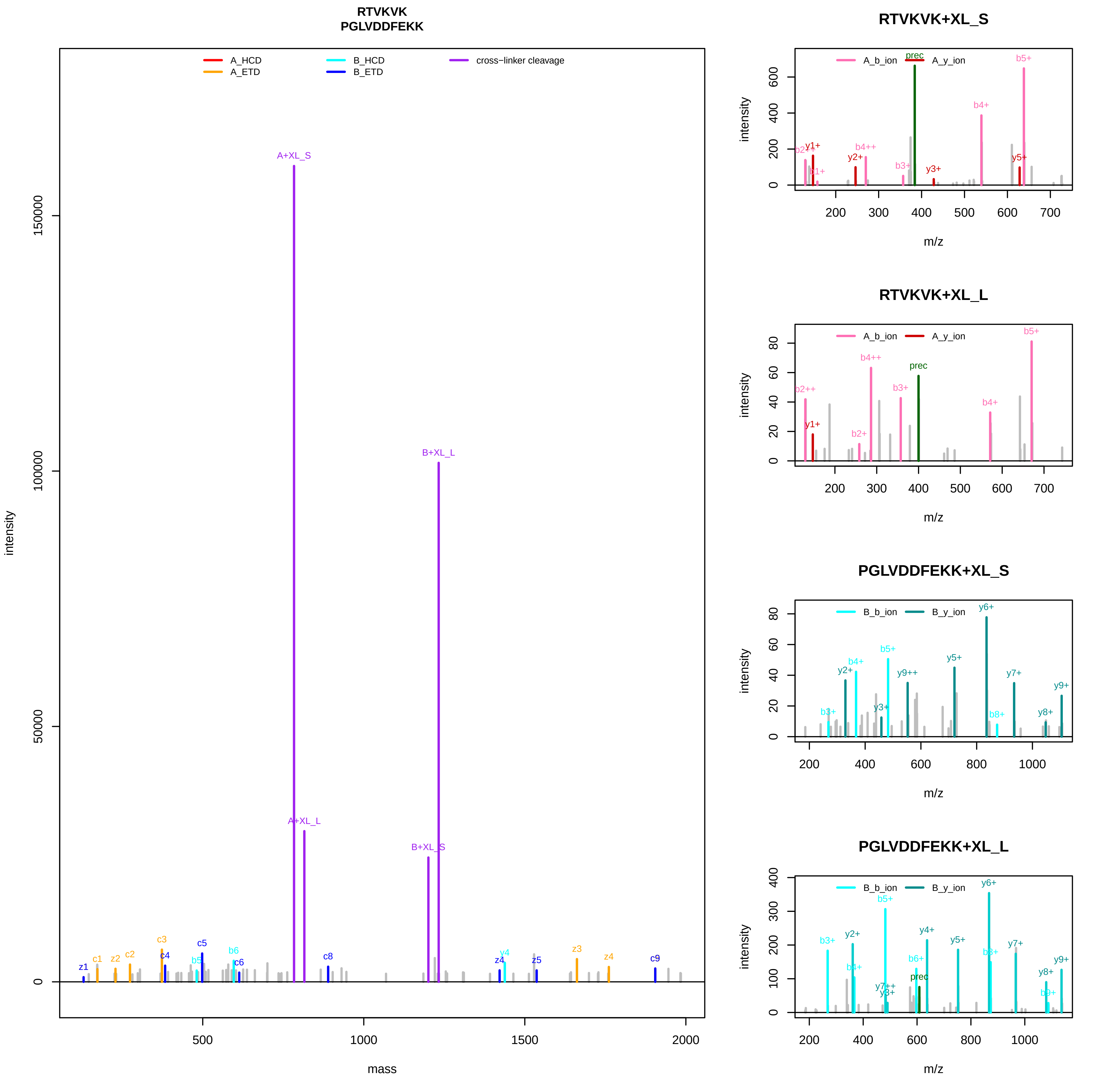
RTVKVK
PGLVDDFEKK
A_HCD
A_ETD
B_HCD
B_ETD
cross−linker cleavage
A+XL_S
B+XL_L
A+XL_L
B+XL_S
intensity
mass
0
50000
100000
150000
500
1000
1500
2000
z1
c1
z2
c2
c3
c4
b5
c5
b6
c6
c8
z4
y4
z5
z3
z4
c9
RTVKVK+XL_S
A_b_ion
A_y_ion
prec
b5+
b4+
b4++
y1+
y2+
b3+
y3+
y5+
RTVKVK+XL_L
b4++
prec
b2++
b3+
b4+
b5+
y1+
b2+
PGLVDDFEKK+XL_S
B_b_ion
B_y_ion
y6+
b5+
b4+
y2+
y5+
y9++
y7+
y9+
b3+
y3+
b8+
y8+
PGLVDDFEKK+XL_L
y6+
b5+
y2+
y4+
b3+
y5+
y7+
b8+
b6+
y9+
b4+
y8+
prec
y7++
y3+
m/z
intensity
